# 56 Implementing sensor-based physical activity surveillance to improve public health policy in The Netherlands: a protocol

**DOI:** 10.1093/eurpub/ckae114.080

**Published:** 2024-09-26

**Authors:** Barbara Snoeker, Hidde van der Ploeg, Maaike Kompier, Wanda Wendel-Vos

**Affiliations:** National Institute for Public Health, Bilthoven, The Netherlands; Amsterdam University Medical Centres, Amsterdam, The Netherlands; CBS (Statistics Bureau Netherlands), Heerlen, The Netherlands; National Institute for Public Health, Bilthoven, The Netherlands

## Abstract

**Purpose:**

Subjective measurement with questionnaires to monitor physical behaviour often leads to recall and social desirability bias. Also, questionnaires are less able to measure relevant aspects of physical activity (PA), such as PA patterns, light intensity PA, and sedentary time. Several Dutch studies have examined the possibility to implement sensor-based monitoring as part of the existing national surveillance infrastructure for PA. These pilots have provided valuable knowledge, but there are still questions about recruitment, implementation logistics, and achieving representativeness of the entire Dutch population. These topics are crucial for feasibility of implementation of monitoring with accelerometers. Therefore, we investigate how accelerometers could best be implemented in future national surveillance systems.

**Methods:**

For this pilot, we use the existing infrastructure from the GGD Health Monitor (GEMON), which is a probability sample from the Dutch population and contains the SQUASH PA questionnaire. At the end of the GEMON, either conducted with a web-based or personal approach, we ask respondents whether they are also willing to participate in our accelerometer pilot. Statistics Netherlands (CBS) selects a random sample of willing respondents (n = 1,000), divided over three socio-economic groups (low, middle, high). Participants wear the activity tracker (ActivPal) for 7 consecutive days. ActivPal data will be linked to the questionnaire data. The primary goal is to recruit a representative sample, and to maximise measurement logistics, data handling and storage. Our secondary goal is to estimate differences in the effectiveness of the approach strategies on response bias and PA behaviour between three socio-economic groups.

**Results:**

For our primary goal, a roadmap will be created in which the web-based as well as the personal approach will be included. For our secondary goal, we determine the most representative recruitment strategy with regard to socio-demographic characteristics and PA behaviour.

**Conclusions:**

Based on the results of the pilot, we advise the Ministry of Health, Welfare and Sports of the Netherlands how to best implement sensor-based PA measurement in the existing national surveillance infrastructure.

**Support/Funding Source:**

This research was funded by the National Institute for Public Health and the Environment, and the Ministry of Health, Welfare and Sports of the Netherlands.

